# Clinical and economic benefit of HPV-load testing in follow-up and management of women postcone biopsy for CIN2–3

**DOI:** 10.1038/sj.bjc.6601032

**Published:** 2003-07-01

**Authors:** B Almog, R Gamzu, J Bornstein, I Levin, O Fainaru, J Niv, J B Lessing, A Bar-Am

**Affiliations:** 1 Cervical Pathology Unit, Department of Obstetrics and Gynecology, Lis Maternity Hospital, Tel Aviv Sourasky Medical Center affiliated to the Sackler Faculty of Medicine, Tel Aviv University, Tel Aviv, Israel; 2 Department of Obstetrics and Gynecology, Cervical Pathology Unit, Carmel Medical Center, Haifa, Israel

**Keywords:** cervical intraepithelial neoplasia, colposcopy, cost – benefit analysis, cytology, histology, human papilloma virus load

## Abstract

This study aimed to evaluate the clinical and economic implications of integrating human papilloma virus (HPV) load testing into the follow-up and management protocol of women postconisation for high-grade cervical intraepithelial neoplasia (CIN2–3). We evaluated 130 suitable women: 63 were screened biannually by Pap smears (‘conventional approach’) and 67 also had HPV-load testing (‘HPV approach’). More stringent criteria for undergoing colposcopy or reconisation were observed by the former group compared to the latter. Both approaches were analysed for cost effectiveness. There were 33 out of 67 (49.2%) colposcopic referrals and 24 out of 67 (35.8%) reconisation/hysterectomies with the ‘conventional approach’ compared to 9 out of 63 (14.2%) and 7 out of 63 (11.1%) with the ‘HPV approach’. Cervical intraepithelial neoplasia 2–3 residual disease was detected in 7 out of 67 (10.5%) and 7 out of 63 (11.1%) women. The ‘conventional approach’ had more negative colposcopic biopsies and more negative reconisation/hysterectomy histologies than the ‘HPV approach’. The respective cost per detection of one case of residual disease was US$3573 and US$3485. The ‘HPV approach’ required fewer colposcopic and reconisation procedures to detect one case of residual CIN2–3. Its higher positive predictive value than that of cytology provided a significant decrease in false positive rates and a reduction of US$88 per detected case.

The diagnosis of high-grade cervical intraepithelial neoplasia (CIN2–3) bears a significant risk of developing invasive carcinoma if not treated (Pinto and Crum, 2000). The recurrence rate after cone biopsy in such cases is estimated to be between 5 and 30% (Bjerre *et al*, 1976; Kolstad and Klem, 1976; Larsson *et al*, 1983). Early diagnosis of residual or recurrent cervical disease is one of the main objectives in the follow-up of women following cone biopsy due to CIN2–3. Three diagnostic modalities, cervical cytology, colposcopy-directed biopsy and human papilloma virus status, have been previously suggested for the follow-up of such women. Currently, cervical cytology is the main screening modality being used for triaging them for further evaluation and treatment (Montz, 2000).

Cervical cytology has significant inherent potential drawbacks. The first and most significant of them is a false negative result which precludes women with undiagnosed lesions from being referred for a proper evaluation, with the consequent obvious risk of developing invasive cervical disease. In addition, cervical cytology is associated with a substantial false positive rate that may create an overload in the work schedule of the limited available colposcopic units, causing a delay in the referral of true positive cases for colposcopic evaluation and leading to over-treatment, which is inevitably accompanied by anxiety, morbidity and monetary costs (Beutner *et al*, 1998).

Colposcopy-directed biopsy is considered as the ‘gold-standard’ diagnostic modality for detecting residual disease after therapeutic conisation. Most recent reports show that the majority of low-grade cervical intraepithelial neoplasia (CIN1 lesions) will spontaneously remit (Baron and Richart, 1970; Noumoff, 1987; Hoskins *et al*, 1992) and that the risk of progression to CIN3 or invasive disease is minor. Accordingly, the main objective is to detect the CIN2–3 lesion, which has a significant risk to progress to invasive disease. Thus, identifying high-grade lesions in low–grade cytology smears becomes a matter of considerable importance.

Human papilloma virus (HPV), which is the most common viral sexually transmitted disease in the United States (Hoskins *et al*, 1992), has since 1977 been considered an aetiologic agent for cervical cancer (Meisels *et al*, 1977) and is currently unequivocally linked with the development of precancerous lesions of the cervix. High-risk HPV subtypes are associated with the presence of high-grade squamous intraepithelial lesion (HSIL) or CIN2–3 (Baron and Richart, 1970). Adding HPV testing to cytology as part of the follow-up protocol of this high-risk group of patients after they had undergone therapeutic cone biopsy due to CIN2–3 may have good predictive ability. Indeed, ter Harmsel *et al* (1999) concluded that persistent infection with HPV 16 is associated with a higher risk of developing CIN, which is often high grade.

Previous studies on the relation between the quantitative level of HPV DNA and histologic severity of cervical lesions have yielded controversial results. Several studies using the semiquantitative polymerase chain reaction analysis (PCR) have shown a correlation between HSIL and a large amount of HPV-16 DNA in cervical samples and a small amount in samples of a low-grade squamous intraepithelial lesion (LSIL) (Cuzick *et al*, 1992, 1994; Bavin *et al*, 1993). In contrast, two other studies using a Hybrid Capture assay revealed smaller amounts of viral DNA in the cervical samples of women with HSIL compared with LSIL (Farthing *et al*, 1994; Sun *et al*, 1995).

We performed this prospective cohort study in order to test our hypothesis that adding assessment of the HPV load of high-risk types as a triaging tool would enable the reduction of the number of colposcopic and reconisation procedures for the detection of one case of residual CIN2–3. To this end, we compared the clinical and economic implications of two different approaches to the follow-up and management of women postconisation due to CIN2–3 lesions, namely the ‘conventional (cytology) approach’ *vs* the ‘HPV (load) approach’.

## MATERIALS AND METHODS

This prospective study was carried out on a cohort of 139 consecutive patients with CIN2–3 lesions who underwent cone biopsies at the Cervical Pathology Unit of the Tel Aviv Medical Center between January 1994 and December 1998. A Pap smear had been used as the screening method for the first 72 patients (Group 1, the ‘conventional approach’) until October 1996, when HPV load assessment was added to the screening protocol of the remaining 67 patients (Group 2, the ‘HPV approach’). The mean follow-up was 53 months (range 25–77). Five of the 72 patients in Group 1 were excluded: three failed to attend all the follow-up visits and the other two were lost to follow-up. Four of the 67 patients in Group 2 were excluded: the HPV test results were incomplete in two and the other two were lost to follow-up.

The mean age was 38.8 years (range 17–54) for the Group 1 patients and 37.4 years (range 16–51) for the Group 2 patients. The following characteristics were similar between the two study groups: contraception use, education level, marital status and positive surgical margins (the latter values were 10.4 and 11.0% for Groups 1 and 2, respectively).

The follow-up protocols as well as the indications for colposcopic and reconisation procedures are summarised in Table 1

**Table 1:** Triaging modalities and indications for colposcopy and reconisation for the two study groups

Procedures	Conventional approach (Group 1)	HPV approach (Group 2)
Triaging modality	Pap smear every 6 months	Pap smear and HPV load every 6 months
Indications for colposcopy	Two consecutive abnormal Pap smears	HSIL in Pap smear or high viral HPV load
Indications for reconisation	Any CIN	CIN2–3

HPV=human papilloma virus; HSIL=high-grade squamous intraepithelial lesion; CIN1, 2–3=cervical intraepithelial neoplasia 1, 2–3.

. The follow-up visits took place at 6-month intervals for up to 2 years and annually thereafter. For Group 1, the indication for colposcopy was two consecutive abnormal Pap smears. The indication for reconisation was a CIN result of any degree on colposcopic biopsy. For Group 2, the indications for colposcopy were HSIL on cytology or a high viral load on an HPV test. The indication for reconisation in this group was restricted to CIN2–3 on colposcopic biopsy. Women with low-grade lesions on cytology (atypical squamous cell of undetermined significance (ASCUS) or LSIL) and a low-load HPV, which did not indicate colposcopy, were followed by serial cytologies (at intervals of 6 months). Likewise, women with low-grade lesions on colposcopy (CIN1) were referred back for follow-up by serial cytology.

We used the type 1 Hybrid Capture Test System (Digene Corporation, Silver Spring, MD, USA). Samples for the presence of a cocktail limited to the following high-risk HPV types (16, 18, 31, 33, 35, 45, 51, 52, 56) were collected from the external cervical os and the exocervix by a special brush applicator and transferred to the lab in a container with transfer media provided in a specific kit by Digene Corp. In the type 1 Hybrid Capture assay, light is emitted during the cleavage of the tested substrate. The intensity of the emitted light is proportional to the amount of DNA present in the examined specimen. A relative light unit (RLU) measurement greater than or equal to the cutoff value indicates the presence of HPV DNA, whereas an RLU measurement less than the cutoff value indicates the absence of an HPV DNA sequence. Based on the above principles, the following scale of HPV load was used: an RLU <0.3 units=a negative HPV DNA test, an RLU between 0.4 and 3 units=a borderline test, an RLU between 4 and 9 units=a low HPV DNA load test and an RLU >10=a high-load HPV DNA test.

A standard cone biopsy was performed by loop excision of the transformation zone (LETZ) followed by laser vaporisation of the crater base and side walls, as previously described (Bar-Am *et al*, 2000). The specimen sizes usually range between 1.8 cm in depth and 1.5 cm in width.

The health insurer of each woman funded the cost of treatment and follow-up. The prices for cost per case of detected residual CIN2–3 and the cost-effectiveness analysis were based on the official prices issued by the country's national healthcare providers: a visit to the doctor=$30, each Pap smear=$18, colposcopy and cervical biopsy=$80, histologic analysis for colposcopy=$50 ($70 for cone histology), Hybrid Capture test=$40 ($70 including doctor's visit) and cone biopsy=$80. These are the government estimates for the cost of the procedures that were issued in order to define tariffs in the public health system (in US dollars according to the rate of exchange for local currency of May 2001). The cost per detected case was calculated accordingly.

### Statistics

Significant differences in proportions were assessed by Fisher's exact test. All the statistical analyses were performed using SPSS for Windows version 8.0 (SPSS Inc., Chicago, IL, USA).

## RESULTS

In all, 67 women who were treated by the ‘conventional approach’ (Group 1) and 63 women who were treated by the ‘HPV approach’ (Group 2) completed the follow-up period according to the study protocol (Table 1). Table 2

**Table 2:** Comparison between the two approaches tested

**Characteristics or outcomes**		**Conventional approach (Group 1) *n*=67**	**HPV approach (Group 2) *n*=63**	*P-*value*
Cytology results (*%*)	LSIL	24 (35.8)	21 (33.3)	0.85
	HSIL	9 (13.5)	6 (9.5)	0.58
				
Procedures performed (*%*)	Colposcopies	33 (49.3)	9 (14.2)	<0.001
	Reconisations/ hysterectomies	24 (35.8)	7 (11.1)	0.001
				
Colposcopic histology (*%*)	Negative	9 (27.2)	1 (11.1)	0.41
	CIN1	17 (51.5)	1 (11.1)	0.03
	CIN2–3	7 (21.2)	7 (77.7)	0.003
				
Reconisation/hysterectomy histology (*%*)	Negative	9 (37.5)	0 (0)	0.07
	CIN1	8 (33.3)	0 (0)	0.09
	CIN2–3	7 (29.2)	7 (100)	0.001
				
Overall CIN2–3 (*%*)		7/67 (10.5)	7/63 (11.1)	1.0

* Significance by Fisher's exact test. HPV=human papilloma virus; CIN1, 2–3=cervical intraepithelial neoplasia 1, 2–3; LSIL=low-grade squamous intraepithelial lesion; HSIL=high-grade squamous intraepithelial lesion.

presents their clinical outcome data. The two groups were equal in terms of the number of LSIL and HSIL cases in each and the overall CIN2–3 residual disease detected by reconisation/hysterectomy.

There were significantly more colposcopic referrals (49.3 *vs* 14.2%, *P*<0.001) and reconisation referrals (35.8 *vs* 11.1%, *P*=0.001) in Group 1 compared to Group 2, respectively. There was a trend of more negative colposcopic biopsies (27.2 *vs* 11.1%) as well as negative reconisation/hysterectomy histologies (37.5 *vs* 0%) in Group 1 compared to Group 2, respectively.

Importantly, there were a total of 22 cases of LSIL or low-load HPV in Group 2 that did not indicate colposcopy, as well as one case of CIN1 on colposcopy. The total number of detected cases of CIN2–3 residual disease was similar between the two groups, that is, 7 (10.5%) and 7 (11.1%) for Groups 1 and 2, respectively.

Table 3

**Table 3:** Positive predictive value and false positive rates of LSIL, HSIL and high-load HPV to any CIN, to CIN1 and to CIN2–3

**Test**	**PPV to any CIN (%)**	**PPV to CIN1 (%)**	**PPV to CIN2–3 (%)**	**False positive rate (%)**
LSIL	33.3	29.1	4.2	66.6
HSIL	77.7	11.1	66.6	22.2
HL-HPV	100	0	100	0

PPV=positive predictive value; CIN1, 2–3=cervical intraepithelial neoplasia grade 1, 2-3; HL=high-load HPV DNA; LSIL=low-grade squamous intraepithelial lesion; HSIL=high-grade squamous intraepithelial lesion.

summarises the precision of prediction of any type of CIN by abnormal cytology or HPV load. The superiority of HPV load in the prediction of any CIN over abnormal cytology can be clearly seen. The positive predictive values (PPV) of LSIL to any CIN were relatively low (4.2–33.3%), whereas the PPV of HSIL to any CIN residual disease or, more importantly, to CIN2–3 was 77.7 and 66.6%, respectively. The PPV of high-load HPV for CIN2–3 was 100%.

The cost of detecting one case of a CIN2–3 lesion according to the ‘conventional approach’ (Group 1) was US$2964. If we had applied the ‘conventional approach’ to the whole study group, the cost-effectiveness ratio would be US$3573. The cost of detecting one case of a CIN2–3 lesion according to the ‘HPV approach’ was US$3485. Thus, adding an HPV load assessment to the standard Pap protocol for this selected group of women would yield a decrease in cost of US$88 per detection of one case of a residual CIN2–3 lesion.

## DISCUSSION

In spite of the relatively high rate of clearance (95% (Reich *et al*, 2002)), patients after conisation due to CIN2–3 are at a higher risk of developing residual or recurrent disease than controls. Their regular follow-up protocol after conisation includes continued cytological and colposcopic examination as well as reconisation in cases of suspected residual/recurrent lesions (Milojkovic, 2002). The optimal management of low-grade cytologies or histologies, such as ASCUS, LSIL or CIN1, in such cases remains undetermined (Livasy *et al*, 1999; Nagai *et al*, 2000; Reich *et al*, 2001).

The main objective of the present prospective cohort study was to analyse the clinical and economic contribution of adding an HPV-load test to the standard cytological follow-up following therapeutic conisation due to CIN2–3. Indications for advanced diagnostic procedures were allowed to be restricted whenever the HPV load was assessed (the ‘HPV approach’), but they were to be expanded whenever only cytology had been used for triaging subjects (the ‘conventional approach’).

A comparison between the two study groups shows that HPV-load evaluation identifies the presence of CIN2–3 at a detection rate equal to the standard cytological approach, but with significantly fewer colposcopies and reconisation procedures. The main contribution of an HPV-load evaluation is its ability to identify normal or low-grade lesions that might progress to high-grade lesions. Three such patients from Group 2 with normal cytologic results were further evaluated only due to the discovery of a high HPV load and they were eventually proved to be CIN2–3. Accordingly, the PPV of a high-load HPV test was maximal (i.e., 100%) according to these findings.

When an HPV-load evaluation is unavailable for the follow-up of such a high-risk group and initial triaging is performed by repeated cytologies, however, one must seriously consider further and complete histological evaluation of all ASCUS, LSIL and CIN1 results. The case of LSIL that was additionally evaluated by colposcopy and subsequently reclassified as CIN2–3 in Group 1 supports this contention. Such a policy, however, results in a high rate of colposcopic and reconisation referrals and, consequently, a high number of low-grade colposcopic and reconisation histologic results.

Previous studies on the relation between the HPV load and the histologic grade of cervical lesions have yielded controversial results. Several investigations in which a semiquantitative PCR was used showed a significant correlation between cytological grade and the amount of HPV-16 DNA in cervical samples (Cuzick *et al*, 1992, 1994; Bavin *et al*, 1993). In contrast, two other studies using Hybrid Capture assays revealed smaller amounts of viral DNA in cervical samples of women with HSIL compared to those with LSIL (Farthing *et al*, 1994; Sun *et al*, 1995). Sun *et al* (2001) recently revealed that the effect of HPV infection on SIL development is dominated by the viral load, thus highlighting a potential application of viral load testing in predicting the advancement of cervical intraepithelial lesions. Ylitalo *et al* (2000) reported that CIN3 is associated with HPV-16-positive women who have consistently high viral loads, and Sherman *et al* (2002) concluded that for women with ASCUS, HPV-load testing is highly sensitive for detecting CIN3 and cancer with dramatically fewer referrals for colposcopy.

We have shown a reduced cost per detection of one case of residual CIN2–3 lesion if HPV-load assessment were to be included in addition to the standard Pap smear. Several authors have independently reported that inclusion of HPV assessment in screening for cervical cancer may be more clinically effective than Pap tests alone at higher but still ‘reasonable costs’ (Goldie *et al*, 2001; Kim *et al*, 2002; Mandelblatt *et al*, 2002). These costs were calculated for quality-of-life measures or years of life saved. Although we did not perform a complete cost-utility analysis, we believe that the distinct significance of the present results applies to the evaluation of different triaging protocols for women at high risk for CIN2–3, rather than as a primary screening protocol. To conclude, we regard the ‘HPV approach’ as the more reasonable choice for such a selective and high-risk group, even had our results shown a small incremental cost.
